# Neonatal hepatitis B vaccination: Reevaluating timing and adjuvants for enhanced safety and effectiveness

**DOI:** 10.1002/pdi3.47

**Published:** 2023-12-11

**Authors:** Aleksandar Cirovic, Ana Cirovic, Ana Ivanovski, Petar Ivanovski

**Affiliations:** ^1^ Institute of Anatomy Faculty of Medicine University of Belgrade Belgrade Serbia; ^2^ Faculty of Medicine University of Belgrade Belgrade Serbia

**Keywords:** aluminum, hepatitis B vaccine, iron deficiency

## Abstract

Our aim is an achievement of a 100 percent vaccination rate without toxic vaccine effects. We did a comprehensive literature review on aluminum neurotoxicity and early neonatal hepatitis B vaccination. We analyzed indications for introduction of early neonatal hepatitis B vaccination and the data on experimental and clinical aluminum‐induced neurotoxicity. It is justified very early hepatitis B vaccination to be carried out in high‐risk endemic areas. In those countries there is a large number of hepatitis B positive individuals, including pregnant women, which leads to a great number of vertical, mother‐to‐child, transmissions of the virus during pregnancy. There is also a serious risk of perinatal Hepatitis B virus infections in such countries. Early neonatal hepatitis B vaccination is also carried out in low‐risk countries, where the number of hepatitis B positive pregnant women is extremely small, making the number of newborns who require the prevention of hepatitis B infection extremely small as well. It is necessary for all vaccines which contain aluminum as an adjuvant to be immediately suspended as children vaccination until they start walking and until aluminum is replaced with calcium, zinc or a nonmetallic adjuvant such as microcrystalline tyrosine or monosodium urate. Meanwhile sustained efforts should be made to create the so‐called mucosal vaccines against diphtheria, pertussis, measles, mumps and rubella. At the same time, all the children born to hepatitis B positive mothers, regardless of their place of birth must be vaccinated against hepatitis B as the benefits from vaccines by far outweigh potential harm caused by adjuvant aluminum.

## 1 CONCISE GENERAL DATA ON HEPATITIS B VIRUS

Hepatitis B virus (HBV) is a small DNA virus whose main feature is pronounced hepatic tropism. Of all the species, only primates and humans could be infected with this virus. HBV is a blood‐borne virus, spherical in shape and when out of the body, it is highly resistant. Crucial parts of HBV are its inner nucleocapsids, enveloped by a lipid layer. HBsAg is the main component of the lipid layer. One of HBV's characteristics is that it replicates via reverse transcription. Until 1970 HBV was referred as Australia antigen.^1^

Viral B hepatitis (VBH) caused by HBV is a global public health problem with an estimated 240 million active infections and the mortality rate comparable to that of HIV, tuberculosis and malaria.^2^, ^3^

Acute form of viral B hepatitis is a common form of the disease with the incubation period which may last between one and six months. Chronic form of VBH leads to liver cirrhosis and hepatocellular carcinoma may occur as well. In the past many jaundice outbreaks were assigned to HBV infection. During the 19th and 20th century, jaundice outbreaks were recorded mainly among shipyard workers and soldiers after being vaccinated against small pox and yellow fever.^4^ Namely, in 1942 one of the largest known epidemics of jaundice occurred and it was caused by parenteral vaccine administration where 50,000 US soldiers were affected,^5^ probably due to the use of nonsterile instruments.

## 2 THE ROUTES OF HBV INFECTION SPREADING

HBV is typically spread among unvaccinated individuals who have multiple sex partners and indulge in casual and unprotected sex. If unvaccinated, viral B hepatitis can also be contracted by injection‐drug users, people who undergo tattooing or piercing or have oral‐dental procedures or surgical procedures done using unsterilized instruments (Table 1).

**TABLE 1:** Evaluation of possibility to get in contact with HBV in newborns and adults.

Potential source of HBV	Probability of newborns to get in contact with HBV	Probability of adults to get in contact with HBV
Oral intervention with unsterilized instruments	‐	+
Tattooing and piercing	‐	+
Drug use with unsterilized needle	‐	+
Blood transfusion	‐	‐
Vertical transmission	+	‐

Abbreviation: HBV, hepatitis B virus.

Newborns of mothers who are positive for both HBsAg and HBeAg get infected during pregnancy by vertical transmission. In endemic areas (central Asian republics, Southeast Asia, Sub‐Saharan Africa and the Amazon basin), beside vertical transmission, newborns can be infected during the perinatal period as well. In underdeveloped countries, the source of VBH may be insufficiently sterilized instruments used during oral surgery and other surgical procedures. Even in the 21st century, hepatitis B infection outbreaks may occur.^6^ Indonesia, for example, is listed as a highly endemic region,^7^ but not all provinces have experienced an equally serious problem with the virus. Namely, the prevalence of hepatitis B surface antigen could differ up to almost 10 times in different provinces in Indonesia.^7^

## 3 HEPATITIS B VACCINE

In 1981, the very first vaccine against HBV was designed and approved in the US. The vaccine was derived from the plasma of those individuals who had been naturally infected with the virus. Upon collection, the plasma underwent ultracentrifugation processes and was treated with formaldehyde, urea and pepsin.^8^ Although highly efficient, this vaccine was abandoned in 1986, because of the constant increase in the number of subjects with HIV. Prior to the invention of the first vaccine, some countries such as Thailand had been considered as hyperendemic.^9^ In the US, a new recombinant DNA vaccine against HBV was approved in 1986 and it has been recommended for routine childhood immunization since 1994.^8^ Regardless of the vaccine manufacturer, 0.25 or 0.5 mg of aluminum (Al^3+^) per dose is an inevitable component of the vaccine against HBV.

## 4 DISCUSSION

There is no doubt that immunization against hepatitis B infection has brought a sharp decrease in the global VBH incidence and consequently a significant decrease in liver cirrhosis and hepatocellular carcinoma. For example, in Thailand, the rate of hepatitis B infection has decreased dramatically since 1992.^3^ The World Health Organization recommended, that is, initiated, worldwide protection from hepatitis B by the introduction of very early neonatal immunization, through vaccine application during the first 6 h upon birth.^10^

It is clear why hepatitis B vaccination is carried out in the neonatal period in high‐risk endemic countries or areas. In those countries, there is a large number of hepatitis B positive individuals, including pregnant women, which leads to a great number of vertical, mother‐to‐child, transmissions of the virus during pregnancy. In addition, there is a serious risk of perinatal HBV infections in such countries. It is, however, rather puzzling that neonatal hepatitis B vaccination is also carried out in low‐risk countries (the US, the EU, and many other countries), where the number of hepatitis B positive pregnant women is extremely small. Consequently, the number of newborns who require prevention from hepatitis B infection‐disease through early neonatal vaccination and possibly the administration of hepatitis B hyperimmunoglobulin is extremely small as well.

In Serbia, every pregnant woman is routinely tested for hepatitis B two to three weeks before giving birth. There are between 60,000 and 70,000 babies born in Serbia each year, and only a few are born to hepatitis B positive mothers. In these cases, those few babies should—or rather must—be protected from hepatitis B through early neonatal vaccination.

However, why are all other newborn babies, who are born to hepatitis B negative mothers, also vaccinated against hepatitis B? How can those newborns in Serbia get infected with hepatitis B?

By receiving infected blood and blood derivatives. This is impossible, as all derivatives are tested and are negative for HBV.
During surgical procedures. This is also impossible as all instruments are HBV free.
During dental procedures, which is not possible as neonates (NN) do not have teeth.
By getting a piercing or tattoo, which is also impossible, as NN do not undergo such procedures.
By being an injection‐drug user. This is also impossible as no neonate has ever been an injection‐drug user before.

In fact, newborns in low‐risk countries will have an opportunity to get in contact with HBV when they reach adulthood and have more chances to make decisions on their own.

On the other hand, numerous experiments have shown that the aluminum presenting in vaccines is neurotoxic^11^ and decreases motor skills development.^12^ There is 0.25 or 0.5 mg of aluminum per dose in hepatitis B vaccines. According to Avogadro's law, 0.25 mg of Al^3+^ has 5.57 × 10^18^ aluminum ions, while 0.5 mg Al^3+^ has 1.11 × 10^19^ aluminum ions. The body of an NN has fewer than 10^13^ cells, which means that upon the administration of the vaccine containing 0.25 mg of Al^3+^ in the first 6 h upon birth, more than 100,000 aluminum ions will be “floating around” all the newborn's cells (10^18^ Al ions/10^13^ body cells). The cells of the NN's central nervous system (CNS) are undoubtedly at the highest risk due to the fact that the CNS is extremely underdeveloped, and toxicology has clearly shown that the less developed the tissue, the more pronounced the toxic effects of the toxin,^13^ as it has already been discussed.^14^, ^15^ Cells with high density of transferrin receptor 1 (TfR1) uptake most of the circulating Al^3+^, since in circulation aluminum is bound to transferrin (Tf). TfR1 is located on the cell surface and the level of TfR1 is modified by iron status—less iron in body will result in increased TfR1 expression while sufficient levels of iron lead to decreased TfR1 levels. In case of iron deficiency, number of TfR1 rises twofold,^16^ and it is reasonable to hypothesize that in this case, cells uptake Al^3+^ more effectively and faster. So, a large amount of free Al ions, (approximately 100,000 ions of Al^3+^ per human cell) may easily bind to Tf, which is overexpressed when the body level of iron is low and enter the cell, leading to cell damage and possible cell death. The presence of iron deficiency among infants varies in rate and could be up to 21%.^17^ In the US, there are about 3,500,000 newborns which means that almost 900,000 newborns could experience iron deficiency during infancy. Many authors analyzed main sources of aluminum in infants (vaccines, breastfeeding and infant formula) and concluded that the cumulative dose of aluminum was beneath the minimal risk level^18^, ^19^ but not far from it. Therefore, if Al toxicity could be potentiated by any condition (e.g., iron deficiency), then a large number of infants could be in unattended danger. A countless number of lives have been saved with hepatitis B vaccine, but choosing a “wrong” moment (i.e., early neonatal period) for vaccine application with a “wrong” adjuvant (i.e., aluminum) could be harmful to all infants, especially those with iron deficiency.

In addition to experimental proof of aluminum neurotoxicity, there are at least two clinical papers dealing with aluminum toxicity to the brain of premature babies^20^ and adults.^21^

Based on all that has been mentioned above, it can be concluded that the vaccination of children against hepatitis B in the neonatal period, if born to hepatitis B negative mothers in low‐risk countries and regions of the world, has neither a medical indication nor a medical justification. In accordance with these, there is a recent study which showed that US male NN vaccinated with hepatitis B vaccine incurred threefold greater risk for autism diagnosis, compared to boys not vaccinated as NN.^22^

Taking the aforementioned into account, we propose, or rather demand, the immediate cessation of hepatitis B vaccination of all babies born to hepatitis B negative mothers in all low‐risk countries and regions.

Moreover, once again and now for the fourth time we call for the immediate suspension of all vaccination with vaccines that contain aluminum as an adjuvant for children aged up to 12 months, that is until the child starts walking and until scientists replace aluminum with calcium (as was the practice in France before 1985) or with zinc or a nonmetallic adjuvant such as microcrystalline tyrosine or monosodium urate.^14^, ^15^, ^23^ At the same time, sustained efforts should be made to create so‐called mucosal vaccines against diphtheria, pertussis,^24^ measles, mumps and rubella. Meanwhile, all the children born to hepatitis B positive mothers, regardless of their place of birth (i.e., low risk or high‐risk countries) must be vaccinated against hepatitis B, even with the current vaccines, as the benefits from vaccines by far outweigh potential harm caused by adjuvant aluminum.

## AUTHOR CONTRIBUTIONS

Conceptualization: Aleksandar Cirovic, Petar Ivanovski. Writing original draft: Aleksandar Cirovic, Petar Ivanovski. Critically revised the work: Ana Cirovic, Ana Ivanovski. Validation: All the Authors.

## CONFLICT OF INTEREST STATEMENT

Authors declare no conflict of interest.

## ETHICS STATEMENT

N/A.

## PATIENT CONSENT STATEMENT

N/A.

## PERMISSION TO REPRODUCE MATERIAL FROM OTHER SOURCES

N/A.

## CLINICAL TRIAL REGISTRATION

N/A.

## Data Availability

The data that support the findings of this study are openly available on PuBmed at https://pubmed.ncbi.nlm.nih.gov/.
